# Editorial: Chromatin modifications and gene expression: from mechanisms to therapeutic implications in disease

**DOI:** 10.3389/fgene.2026.1835991

**Published:** 2026-04-10

**Authors:** Liliana Burlibasa, Giovanni Nassa, Anca Botezatu, Annamaria Salvati

**Affiliations:** 1 Department of Genetics, Faculty of Biology, University of Bucharest, Bucharest, Romania; 2 Department of Medicine, Surgery and Dentistry “Scuola Medica Salernitana”, University of Salerno, Baronissi, Italy; 3 Genome research center for Health-CRGS, Salerno, Italy; 4 Molecular Pathology and Medical Genomic Unit, University Hospital “San Giovanni di Dio e Ruggi d’Aragona”, Salerno, Italy; 5 Department of Molecular Virology, “Stefan S. Nicolau” Institute of Virology, Bucharest, Romania

**Keywords:** biomarkers, chromatin remodeling, epigenetic mechanisms, gene expression, gene regulation, microRNAs, therapeutic targets

Gene expression is a complex process controlled by an interplay of different regulatory factors that reach far beyond the simple DNA sequence. Epigenetic mechanisms, chromatin architecture, and regulatory RNA molecules are the key components of the molecular networks that coordinate cellular identity, physiological adaptation, and pathological conditions. Over the past decade, advances in high-throughput sequencing technologies, epigenomic profiling, and computational tools have transformed our understanding of these regulatory processes, revealing intricate molecular interactions that operate across genomic, transcriptomic, and epigenomic levels.

The Research Topic *Chromatin Modification and Gene Expression: From Mechanisms to Therapeutic Implications in Disease* brings together a collection of articles focusing on the complex roles of epigenetic regulation and non-coding RNAs in a variety of biological contexts. Collectively, the contributions highlight how regulatory RNAs, chromatin architecture, and epigenetic modifications interact to shape the gene expression programs and influence disease initiation and progression. Importantly, the articles emphasize the value of integrative approaches that combine experimental and computational strategies to better understand the sophisticated regulatory landscape. Moreover, increasing evidence indicates that disruptions in these chromatin-associated regulatory systems contribute to the development of numerous human diseases, including cancer, cardiovascular disorders, inflammatory conditions, and developmental abnormalities.

One of the central themes addressed in the published articles is the role of non-coding RNAs, particularly microRNAs, in modulation of gene expression. MicroRNAs and other classes of regulatory RNAs act as fine-tuning elements within gene regulatory networks, influencing complex processes ranging from immune response and inflammation to cellular differentiation and tissue homeostasis. For example, the role of miR-425-5p in immune-mediated diseases and inflammatory responses is highlighted in the study performed by Zhou et al. This study highlights how microRNA-mediated regulation contributes to the molecular mechanisms underlying autoimmune myocarditis. Similarly, the broader biological implications of miR-425 dysregulation and its potential diagnostic and therapeutic applications are reviewed in the work of Zhou and Han. These studies further reinforce the concept that microRNAs serve not only as mechanistic regulators, but also as potential biomarkers for diagnostic, prognostic, and therapeutic targets in complex diseases, emphasizing the growing relevance of non-coding RNA–mediated regulatory pathways in cardiovascular pathology.

Another important direction addressed in this collection concerns the influence of chromatin structure and epigenetic modifications on gene regulatory programs. Chromatin organization plays a critical role in controlling access to genetic information and coordinating the transcriptional activity. Histone modifications, DNA methylation patterns, and chromatin remodeling complexes contribute to the establishment of a dynamic transcriptional state that enables cells to respond to environmental signals. The functional role of chromatin-associated regulatory proteins is illustrated by Liu et al., providing an in-depth analysis of PHF23, and offers insights into how chromatin reader factors participate in transcriptional regulation and disease-related signaling pathways. Notably, PHF23 can function either as a tumour suppressor or as an oncogenic factor depending on cellular context, highlighting the complexity of chromatin-reader proteins in gene regulation. In addition, the review by Cheng et al. highlights the role of super-enhancers as key drivers of gene regulatory networks, emphasizing their contribution to transcriptional control in cancer and other complex diseases.

Recent progress in understanding the three-dimensional organization of the genome has also provided new insights into gene regulation. In this Research Topic, Reyes-Gopar et al. demonstrate how chromosome conformation studies and gene expression analyses can uncover regulatory interactions that shape transcriptional landscapes. Such approaches highlight the importance of considering genome organization as an integral component of gene regulatory systems during tumorigenesis.

Beyond chromatin, another important layer of epigenetic control is represented by extracellular communication mediated by regulatory RNAs, and extracellular vesicles. Intercellular signaling through vesicle-associated microRNAs can influence developmental processes, tissue homeostasis, and disease progression by modulating gene expression. The study performed by Cao et al., investigating extracellular vesicles involved in mandibular morphogenesis, illustrates how vesicle-mediated RNA transfer contributes to tissue development and intercellular communication, emphasizing the role of epigenetic signaling beyond individual cells and tissues.

The Research Topic also reflects the increasing importance of integrative approaches in modern genetics and genomics. Complex analyses that combine multiple types of biological data are becoming essential for deciphering the complexity of gene regulation. Computation network models, multi-omics integration, and bioinformatics–driven discovery strategies are enabling researchers to identify new biomarkers, explore regulatory pathways, and generate hypotheses that can subsequently be validated experimentally. For example, Guo et al., through transcriptomic analysis combined with network-modeling and machine-learning approaches, identify macrophage-associated biomarkers linked to regulatory pathways. Such integrative frameworks are particularly valuable in the study of multifactorial diseases, where genetic, epigenetic, and environmental factors interact to shape disease outcomes.

**FIGURE 1:**
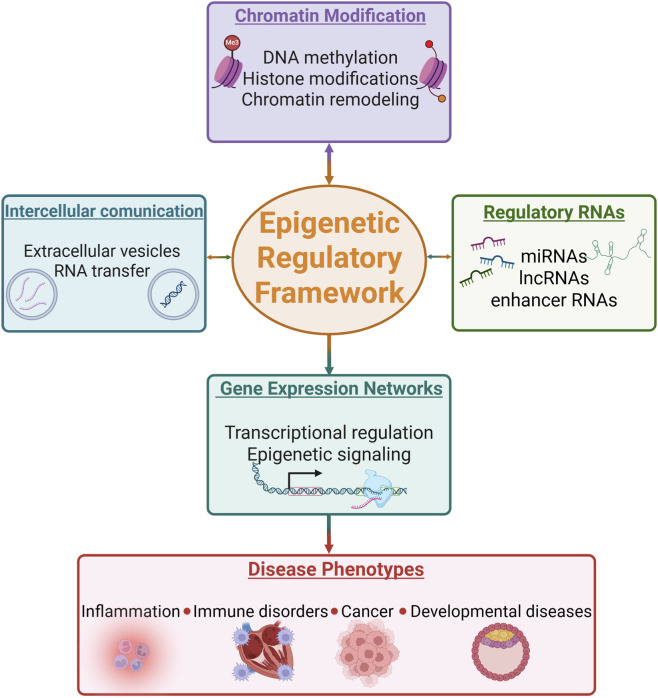
Chromatin modifications and regulatory RNAs shaping gene expression and disease. *Created in BioRender. Burlibasa, L. (2026)*.

Altogether, the articles published in this Research Topic provide a snapshot of the current progress made in the field of epigenetic regulation and gene expression, orchestrated through the coordinated action of multiple chromatin-associated regulatory layers (Figure 1). While considerable advances have been achieved, the regulatory networks that control cellular function remain highly complex. Continued efforts to integrate molecular, genomic, and bioinformatics approaches will be essential for unraveling these networks and for translating fundamental discoveries into clinically relevant applications.

Looking forward, the development of single-cell technologies, spatial transcriptomics, and advanced epigenomic profiling methods will provide new opportunities to study gene regulation with improved resolution and precision. These tools are expected to reveal new aspects of chromatin organization, regulatory RNA function, and epigenetic signaling that are still poorly understood. Moreover, translating these discoveries into clinical applications will contribute to developing novel diagnostic and targeted therapeutic strategies.

In conclusion, this Research Topic provides an overview of current progress in the field of epigenetic regulation and regulatory RNAs, highlighting the importance of integrative approaches to studying and understanding complex biological systems. The review articles found in the Research Topic help to drive forward our knowledge about the role of epigenetics in immunology, pathogenesis, and possible clinical intervention of complex diseases. We are hopeful that the insights presented here can stimulate further research aimed at deciphering the intricate regulatory networks that shape cellular function and human health.

